# Exploratory Characterization of Coronary Thrombi by Integrated Mass Spectrometry and Elemental Imaging in Acute Coronary Syndrome

**DOI:** 10.7759/cureus.103275

**Published:** 2026-02-09

**Authors:** Mayo Wada, Tadayuki Ogawa, Setsu Nishino, Ryota Hashimoto, Masashi Sakuma, Shigeru Toyoda

**Affiliations:** 1 Department of Cardiovascular Medicine, Dokkyo Medical University, Mibu, JPN; 2 Laboratory for Molecular Pathobiology, Research Center for Advanced Medical Science, Dokkyo Medical University, Mibu, JPN

**Keywords:** acute coronary syndrome (acs), coronary thrombus, elemental imaging, lipid profiling, mass spectrometry imaging, molecular pathology, plaque erosion, plaque rupture, sem-eds, thrombus composition

## Abstract

Background

The molecular composition of coronary thrombi varies according to the underlying pathophysiology of acute coronary syndrome (ACS); however, exploratory, spatially resolved analyses integrating molecular and elemental information across intravascular ultrasound (IVUS)-defined thrombus types remain limited.

Objectives

This study aims to exploratorily and descriptively characterize, as a proof-of-concept, thrombus-type-specific molecular and elemental features by combining matrix-assisted laser desorption/ionization mass spectrometry imaging (MALDI-MSI) with scanning electron microscopy and energy-dispersive X-ray spectroscopy (SEM/EDS), and to assess associations with clinical background factors, including statin exposure as a contextual modifier rather than an intervention.

Methods

A total of 11 coronary thrombi aspirated during percutaneous coronary intervention (PCI) for ACS were collected consecutively, and seven representative samples were analyzed. Thrombi were classified into five IVUS-based categories: plaque rupture (PR), lotus-root-like organized thrombus (LR), calcified nodule (CN), thromboembolism (TE), and plaque erosion (E). Serial cryosections were subjected to MALDI-MSI in the positive-ion mode (m/z 120-1000) to visualize representative molecules, including cholesterol, phosphatidylcholine (PC), sphingomyelin (SM), flavin mononucleotide (FMN), and heme b. Adjacent sections were evaluated by SEM/EDS to assess thrombus structure and elemental distribution.

Results

Cholesterol was detected in all thrombus types. Within PR lesions, higher cholesterol signal intensities were observed in statin-naïve cases compared with statin-treated cases, representing an observed association rather than evidence of direct therapeutic modulation. LR lesions exhibited relatively low cholesterol signals despite statin-naïve status, consistent with characteristics of more organized thrombi. In contrast, TE and E lesions showed abundant and diffusely distributed cholesterol irrespective of statin exposure. PC and SM displayed heterogeneous distribution patterns across thrombus types, with PC prominently detected in LR lesions and variable SM signals across clinical backgrounds. Heme b was strongly detected in PR and TE, in line with erythrocyte-rich thrombi, whereas LR and CN showed low levels compatible with platelet-dominant compositions. FMN showed heterogeneous patterns across thrombus types, which should be regarded as hypothesis-generating. SEM/EDS revealed pathology-consistent findings, including prominent calcium accumulation in CN and distinct structural architectures corresponding to IVUS-based classifications. Exploratory principal component analysis of MSI data suggested overall patterns of variability across thrombus types, without inferential or classificatory interpretation.

Conclusions

Integrated molecular and elemental imaging revealed heterogeneous, thrombus-type-specific features across intravascular ultrasound-defined acute coronary syndrome phenotypes, representing descriptive, spatially resolved associations rather than causal relationships. This exploratory, hypothesis-generating multimodal approach using MALDI-MSI and SEM/EDS provides an initial framework for characterizing coronary thrombi in situ, with further validation in larger, prospectively enrolled cohorts required to determine potential clinical relevance.

## Introduction

Acute coronary syndrome (ACS) is a major cardiovascular condition caused primarily by thrombus formation following plaque rupture (PR) or erosion (E), leading to life-threatening cardiac events [1-3]. ACS occurs when disruption of an atherosclerotic plaque results in rapid luminal narrowing or occlusion of the coronary artery, ultimately progressing from myocardial ischemia to infarction [1]. Clinically, ACS is categorized into ST-segment elevation myocardial infarction (STEMI), non-ST-segment elevation myocardial infarction (NSTEMI), and unstable angina (UA), based on electrocardiographic findings and the presence or absence of biomarkers of myocardial necrosis.

STEMI is defined by prolonged chest pain or pressure lasting more than 20 minutes, ST-segment elevation in at least two contiguous leads or new-onset left bundle branch block, and a rise and/or fall in high-sensitivity troponin levels. NSTEMI presents with similar ischemic symptoms but without ST-segment elevation; ST-segment depression or T-wave inversion may be observed, and myocardial necrosis is confirmed by elevated troponin levels. UA is characterized by chest pain at rest or with minimal exertion, often with increasing frequency or severity, without troponin elevation; electrocardiographic changes are transient or may be absent.

Advances in pharmacological therapy, including statin treatment, as well as improvements in percutaneous coronary intervention (PCI), have substantially improved the prognosis of ACS. Nevertheless, patients remain at considerable risk of recurrent ischemic events and adverse outcomes, highlighting the need for improved risk stratification and more individualized therapeutic approaches. Conventional lipid management has largely focused on reducing circulating low-density lipoprotein (LDL) cholesterol levels, and lipid-lowering therapies such as statins are well established to reduce cardiovascular events [4,5]. However, the pathogenesis of ACS involves a complex and heterogeneous cascade of molecular processes during thrombus formation, including alterations in lipid composition, inflammatory activation, oxidative stress, and calcium deposition. Importantly, ACS continues to occur in a subset of patients despite ongoing statin therapy, suggesting variability in the molecular mechanisms underlying plaque destabilization and thrombogenesis [6]. Despite this complexity, relatively few studies have comprehensively examined the molecular characteristics of coronary thrombi in relation to clinical and imaging features [7,8]. In contrast, imaging modalities such as coronary angiography (CAG), intravascular ultrasound (IVUS), and optical coherence tomography (OCT) are well established for characterizing the location and morphology of culprit lesions [9], underscoring the potential value of integrating imaging-based lesion assessment with spatially resolved molecular analyses.

In this exploratory study, we analyzed coronary thrombi aspirated during PCI in patients with ACS using matrix-assisted laser desorption/ionization mass spectrometry imaging (MALDI-MSI) and scanning electron microscopy coupled with energy-dispersive X-ray spectroscopy (SEM/EDS) to visualize pathology-associated molecular and elemental features. MALDI-MSI enables high-resolution spatial mapping of molecular distributions within tissue sections and is particularly suitable for thrombus analysis, as it allows simultaneous detection of multiple lipids and metabolites implicated in cardiovascular disease. SEM/EDS provides complementary information on thrombus microstructure and elemental composition [8].

Aspirated coronary thrombi were classified into five categories: plaque rupture (PR), lotus-root-like structure (lotus root (LR)), calcified nodule (CN), thromboembolism (TE), and plaque erosion (E) [10]. Using MALDI-MSI in the positive-ion mode, we characterized the spatial distributions of representative molecules, including cholesterol, phosphatidylcholine (PC), sphingomyelin (SM), flavin mononucleotide (FMN), and heme b. Structural characteristics and elemental distributions were further evaluated using SEM/EDS. By integrating these molecular and elemental findings with clinical and imaging information, this study aims to provide an initial descriptive framework of pathology-specific features of coronary thrombi and to generate hypotheses for future validation in larger cohorts, rather than to infer causal relationships, including those related to statin therapy.

## Materials and methods

Patients and thrombus collection

This study was approved by the Institutional Review Board of Dokkyo Medical University Hospital (approval number: R-80-2J), and written informed consent was obtained from all enrolled patients. The study population consisted of patients with acute coronary syndrome (ACS) who underwent percutaneous coronary intervention (PCI) at the Department of Cardiovascular Medicine, Dokkyo Medical University.

The diagnosis of ACS and its subtypes was established according to contemporary international clinical guidelines, based on a comprehensive assessment of clinical presentation, electrocardiographic findings, and cardiac biomarker measurements. Thrombus aspiration was performed at the discretion of the attending interventional cardiologist during the index PCI, typically in the acute phase following symptom onset. Coronary thrombi aspirated using a dedicated aspiration catheter during PCI were collected consecutively and immediately stored at -80°C until analysis. A target sample size of at least 10 thrombi was predefined for this exploratory study. In total, 11 thrombi were collected. Of these, seven representative samples were selected for integrated matrix-assisted laser desorption/ionization mass spectrometry imaging (MALDI-MSI) and scanning electron microscopy/energy-dispersive X-ray spectroscopy (SEM/EDS) analyses based on predefined technical and morphological criteria. These criteria included sufficient tissue volume, preservation of thrombus architecture, minimal fragmentation during aspiration, and feasibility of serial cryosectioning across multiple analytical modalities. The remaining samples were excluded from integrated analyses due to insufficient tissue integrity or limitations in serial section preparation.

For each patient, clinical information, including age, sex, cardiovascular risk factors, and medication history (e.g., statins and antiplatelet agents), was obtained from medical records. Statin exposure was defined based on medication information documented at the time of hospital admission. Specifically, statin use at presentation (yes/no), statin type, and prescribed daily dose were recorded. Because many patients with ACS were transferred from community hospitals, detailed information regarding the exact initiation date and duration of statin therapy prior to admission could not be reliably ascertained. Therefore, cumulative statin exposure was not quantitatively assessed, and treatment history was recorded descriptively. Peripheral blood parameters measured at hospital admission included total cholesterol, low-density lipoprotein cholesterol (LDL-C), high-density lipoprotein cholesterol (HDL-C), hemoglobin (Hb), and C-reactive protein (CRP). Imaging evaluation consisted of coronary angiography (CAG), intravascular ultrasound (IVUS; AltaView, Terumo Corporation, Tokyo, Japan; OptiCross, Boston Scientific Corporation, Marlborough, MA), and optical coherence tomography (OCT; Dragonfly catheter, Abbott Vascular, Santa Clara, CA) to assess the anatomical location and morphological characteristics of the culprit lesions [9]. Clinical time-course variables were collected from medical records, including the interval from symptom onset to coronary reperfusion, defined as the time from symptom onset to first balloon inflation during PCI (onset-to-balloon time). In cases in which the exact time of symptom onset was unclear, this interval was estimated based on available clinical history and documentation. Given the heterogeneity of this variable and the exploratory nature of the study, onset-to-balloon time was summarized descriptively and was not used as a stratification factor in molecular or elemental analyses.

Thrombus pathology classification

Using intravascular imaging, culprit lesion phenotypes were classified into five pathological categories: plaque rupture (PR), lotus-root-like structure (lotus root (LR)), calcified nodule (CN), thromboembolism (TE), and plaque erosion (E). PR, LR, and CN were identified based on IVUS findings, whereas E was assessed using OCT. Three experienced analysts independently reviewed the IVUS and OCT images while blinded to clinical data and molecular results. Any discrepancies were resolved through consensus discussion. Formal inter-observer agreement metrics were not calculated due to the limited cohort size and the exploratory nature of the study. PR was defined as the disruption of the fibrous cap with exposure of the lipid core, accompanied by fresh thrombus formation, representing a typical ACS phenotype. LR was characterized by a multi-channel morphology, reflecting chronic organized lesions with repeated cycles of recanalization and re-occlusion. Calcified nodules were defined as protruding, irregular calcified masses extending into the lumen with acoustic shadowing. TE was defined as the presence of culprit-lumen thrombus without intravascular imaging evidence of an underlying atherosclerotic culprit phenotype, including plaque rupture, plaque erosion, or calcified nodule. The diagnosis of TE was supported by angiographic and/or clinical features suggestive of coronary embolism. E was defined on OCT as attached thrombus over an intact fibrous cap without evidence of cap disruption or cavity formation. In addition to imaging-based classification, macroscopic examination of aspirated material was performed to categorize thrombi as red thrombi (erythrocyte- and fibrin-rich), white thrombi (platelet-dominant), or mixed-type thrombi. Formal inter-observer variability statistics were not calculated because of the limited sample size; this limitation is acknowledged in the Discussion.

Tissue preparation and morphological evaluation

**Figure 1:**
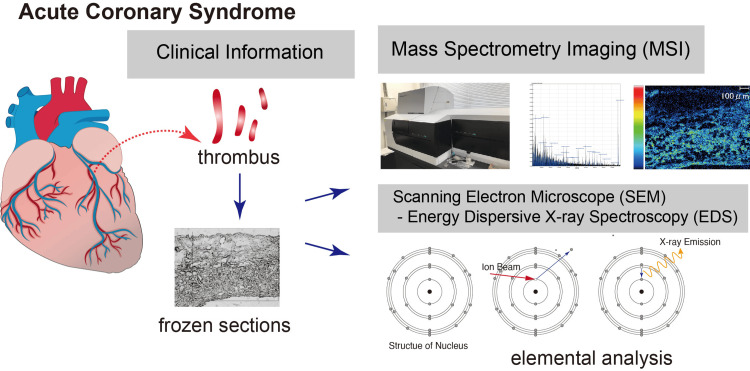
Overall workflow of sample collection, imaging evaluation, and molecular analyses of aspirated coronary thrombi This figure illustrates the experimental workflow used in this study. Aspirated coronary thrombi were collected during primary PCI, followed by imaging evaluation of the culprit lesions with CAG, IVUS, and OCT. Each sample subsequently underwent integrated analyses, including MALDI-MSI for molecular profiling and SEM/EDS for structural and elemental characterization. The workflow summarizes the step-by-step procedures used to correlate imaging-based lesion phenotypes with molecular and elemental signatures. This figure was originally created by the authors, and no external permissions were required. PCI: percutaneous coronary intervention, CAG: coronary angiography, IVUS: intravascular ultrasound, OCT: optical coherence tomography, MALDI-MSI: matrix-assisted laser desorption/ionization mass spectrometry imaging, SEM/EDS: scanning electron microscopy/energy-dispersive X-ray spectroscopy

Aspirated thrombi were stored at -80°C until analysis. At the time of analysis, serial cryosections with a thickness of 10 µm were prepared using a cryostat (Leica CM3050 S, Leica Microsystems GmbH, Nussloch, Germany). Selected sections were stained with hematoxylin and eosin (HE) and examined by light microscopy to evaluate cellular components and structural characteristics. Adjacent serial sections from the same tissue block were reserved for MALDI-MSI and SEM/EDS analyses to enable spatial comparison across modalities (Figure 1).

Sample preparation and mass spectrometry imaging (MALDI-MSI)

Thrombus specimens were embedded in 4% carboxymethyl cellulose (CMC; Fujifilm Wako Pure Chemical, Osaka, Japan) and cryosectioned at a thickness of 10 µm using a cryostat (Leica CM1950) at -20°C. Sections were mounted onto conductive indium tin oxide (ITO)-coated glass slides (SI0100N, Matsunami, Osaka, Japan). As a matrix, 2,5-dihydroxybenzoic acid (DHB; 149357, Sigma-Aldrich, St. Louis, MO) was uniformly applied using an automated matrix deposition system (iMLayer, Shimadzu, Kyoto, Japan). MALDI mass spectrometry imaging (MALDI-MSI) was performed using an iMScope TRIO (Shimadzu) (Figure 1) in the positive-ion mode over an m/z range of 120-1000, with a laser beam size of 5 µm, laser power of 16.2, and a spatial resolution of 5 µm (5 µm × 5 µm pixel pitch). MSI data were processed and visualized using Imaging MS Solution (version 1.30, Shimadzu) and IMAGEREVEAL MSI (version 1.30, Shimadzu) (Figure 1).

Molecular annotations were categorized as either putatively identified or putatively assigned. Putatively identified annotations were based on concordant accurate mass measurements and MS/MS fragmentation patterns obtained from tissue samples and authentic standards. Putatively assigned annotations were based on accurate mass matching, consideration of ion species, and reference to prior literature and database resources. The ions at m/z 369.35 (cholesterol, C₂₇H₄₆O, [M-H₂O+H]⁺) and m/z 457.11 (flavin mononucleotide (FMN), C₁₇H₂₁N₄O₉P, [M+H]⁺) were classified as putatively identified based on close agreement between observed and theoretical masses, as well as matching MS/MS fragmentation patterns acquired from tissue sections and authentic standards (cholesterol: Sigma-Aldrich, C8667-5G; FMN: Nacalai Tesque, 16011-41). These assignments were further supported by consistency with previously reported mass spectrometry imaging studies. The ion at m/z 184.07, corresponding to the phosphatidylcholine headgroup (C₅H₁₄NO₄P, [M+H]⁺), and the ion at m/z 617.18, corresponding to heme b (C₃₄H₃₂FeN₄O₄, [M+H]⁺), were classified as putatively assigned based on accurate mass matching and well-established literature reports describing these ions in biological tissues. The ion at m/z 725.55 was annotated as putatively assigned sphingomyelin (SM 34:1;O₂, C₃₉H₇₉N₂O₆PNa, [M+Na]⁺) based on accurate mass matching, consideration of sodium adduct formation, database searches in Metabolomics Workbench, and consistency with prior reports of sphingomyelin detection in mass spectrometry imaging studies. Accurate mass matching was performed using a tolerance of ±10 ppm, which was uniformly applied in this study. This tolerance was selected to account for matrix-related effects and local ionization variability inherent to atmospheric-pressure MALDI-MSI, particularly for low-molecular-weight ions.

Δppm indicates the signed mass error calculated as (*Observed m/z − Theoretical m/z*) / *Theoretical m/z* × 10⁶. Structural and stereoisomers cannot be completely excluded.

Regions of interest (ROIs) were manually defined based on morphological features to include thrombus-rich areas while excluding background regions. Signal intensities were normalized using total ion current (TIC) normalization to facilitate comparison across tissue sections and samples. Imaging analyses focused on representative detected ions within the m/z 120-1000 range acquired in positive-ion mode, with particular emphasis on molecular species previously reported to be relevant to thrombus and plaque biology, including cholesterol. Because pixel-level intensity distributions in mass spectrometry imaging data were highly non-normal and contained a substantial proportion of zero-intensity pixels, summary statistics are presented primarily as the median and interquartile range (IQR), which provide robust measures under these conditions. Mean values and standard deviations (SDs) are additionally reported to describe the magnitude and variability of non-zero signal intensities. For quantitative comparisons, two ROIs were analyzed per sample whenever feasible, and mean signal intensities were calculated for each ROI. This approach yielded approximately 20,000 pixel-level data points per comparison. Statistical comparisons between ROIs were performed using the non-parametric Mann-Whitney U-test. When the analysis software reported a p-value of 0, results were expressed as p < 0.0001.

Principal component analysis (PCA) was performed using IMAGEREVEAL (Shimadzu) as an unsupervised, non-targeted exploratory approach to visualize global spectral patterns in mass spectrometry imaging (MSI) data. PCA was conducted on spectra acquired over an m/z range of 150-1000, which were binned at 0.2 Da intervals to construct the data matrix. Prior to PCA, spectral intensities were normalized by total ion current (TIC) to account for variability in overall signal intensity across pixels and samples. PCA was performed on ROI-averaged spectra, and Pareto scaling was applied to balance the contributions of high- and low-abundance ions without overemphasizing low-intensity noise. Within MSI-imaged regions, ROIs were placed to include only specimen-derived tissue areas, defined as regions without gaps or voids introduced during section preparation. ROIs were positioned in a quasi-random manner while ensuring morphological consistency. For each sample, three or more ROIs were analyzed whenever tissue size permitted. Due to specimen size limitations, predefined exceptions were applied, whereby one ROI was analyzed for TES- samples and two ROIs for LRS- samples. All ROIs were defined as square regions of fixed size (200 μm × 200 μm) to ensure consistency across samples. The percentage of variance explained by each principal component was calculated and reported. PCA was used exclusively for exploratory visualization of spectral patterns and was not intended for statistical inference or predictive modeling. Given the exploratory nature of the study and the limited sample size, p-values are reported descriptively without formal adjustment for multiple comparisons and are interpreted cautiously.

Scanning electron microscopy and elemental analysis (SEM/EDS)

Serial frozen sections obtained from the same thrombus specimens were mounted on aluminum adhesive tape and examined using a scanning electron microscope (SEM; Hitachi TM3000, Hitachi, Tokyo, Japan) for morphological assessment (Figure 1). Elemental analysis was subsequently performed using a low-vacuum scanning electron microscope (LVSEM; TM3000, Hitachi, Japan) equipped with an energy-dispersive X-ray spectrometer (EDS; Quantax 70, Bruker, Billerica, MA) (Figure 1). SEM images were acquired at 100× magnification with an accelerating voltage of 15.0 kV. The working distance was set to 8.5 ± 0.5 mm. EDS elemental mapping was performed with a total acquisition time of 70 s per field of view. Elemental composition was analyzed from the acquired EDS spectra using the Quantax 70 software (Bruker). The elements analyzed included Na, Mg, P, S, K, Ca, Zn, and Fe, which were selected as representative indicators of elemental accumulation and deposition within thrombus components. Elemental data derived from EDS were interpreted in a semi-quantitative manner. Normalized weight percentages (wt%) were used to compare relative elemental composition across regions analyzed under identical acquisition conditions; however, these values do not represent absolute elemental concentrations. Because EDS measurements in heterogeneous biological specimens are influenced by factors such as section thickness, surface topography, and matrix composition, SEM/EDS findings were interpreted as semi-quantitative and complementary to MSI data. Accordingly, emphasis was placed on relative differences in elemental distribution patterns and spatial concordance with molecular signals observed by MSI, rather than on absolute elemental quantification.

## Results

Macroscopic characteristics of aspirated thrombi and structural classification by IVUS

A total of 11 coronary thrombi were collected from patients with acute coronary syndrome during PCI using an aspiration catheter, of which seven representative samples were selected for integrated analyses. For each patient, clinical background information, including age, sex, and medication history such as lipid-lowering therapy, was recorded, together with peripheral blood biochemical parameters measured at admission (total cholesterol, LDL-C, HDL-C, triglycerides, hemoglobin (Hb), and C-reactive protein (CRP)) (Table 1).

**Table 1:** Clinical characteristics of patients and aspirated coronary thrombi Statin use at presentation was categorized as “S+” (with statin therapy) or “S-” (without statin therapy) for each thrombus type: PR, LR, CN, E, and TE, resulting in designations such as PRS+/PRS-, LRS+/LRS-, CNS+/CNS-, ES+/ES-, and TES+/TES-. Clinical background variables, including age, sex, comorbidities, culprit lesion location, and medication history (statin use at presentation, statin type, statin dose at admission, and history of statin therapy), are summarized for each patient. Statin-treated and statin-naïve status was defined based on medication information available at presentation: statin-treated patients were those receiving any statin therapy at admission, whereas statin-naïve patients had no statin therapy documented. Because many patients were transferred from community hospitals, detailed information on statin duration and adherence prior to admission was unavailable and was not quantitatively assessed. Peripheral blood biochemical parameters measured at admission, including T-Chol, LDL-C, HDL-C, TG, Hb, and CRP, are also presented. Thrombus color features and intravascular imaging findings (IVUS) are shown alongside clinical diagnoses, and thrombi were classified as PR, LR, CN, TE, or E based on coronary angiography and IVUS findings. The time from symptom onset to coronary reperfusion (onset-to-balloon time) was recorded from clinical documentation; when onset timing was unclear, it was estimated retrospectively, sometimes resulting in prolonged intervals. Given its heterogeneity and the limited cohort size, onset-to-balloon time was summarized descriptively and not used for stratification in downstream molecular or elemental analyses. PR: plaque rupture, LR: lotus-root-like lesion, CN: calcified nodule, E: plaque erosion, TE: thromboembolism, T-Chol: total cholesterol, LDL-C: low-density lipoprotein cholesterol, HDL-C: high-density lipoprotein cholesterol, TG: triglycerides, Hb: hemoglobin, CRP: C-reactive protein, IVUS: intravascular ultrasound

Sample	PRS-	PRS+	LRS-	CNS-	TES-	TES+	ES+
Color feature	Mix	Red	White	White	Red	Red	Red
IVUS imaging	PR	PR	LR	CN	TE	TE	E
Age (years)	58	84	55	40	88	80	75
Sex ( male/female)	Male	Male	Male	Female	Male	Male	Male
Diagnosis	STEMI	STEMI	STEMI	STEMI	NSTEMI	NSTEMI	NSTEMI
Comorbidities	CAVB, DLp	DLp	VF	MCTD	Af, CAVB	Paf, DLp	DLp
Culprit lesion	RCA#1	RCA#2	RCA#1	HL	RCA#4	LAD#7	RCA#1
Statin use at presentation	-	+	-	-	-	+	+
Statin type	-	Pitavastatin	-	-	-	Rosuvastatin	Atorvastatin
Statin dose at admission	-	2 mg once daily	-	-	-	5 mg once daily	5 mg once daily
History of statin therapy	-	Unavailable	-	-	-	Available ≧ 1 year	Unavailable
T-Chol (mg/dL)	191	147	205	253	221	191	158
TG (mg/dL)	41	162	77	248	134	119	115
HDL-C (mg/dL)	39	37	38	31	41	65	43
LDL-C (mg/dL)	144	78	152	173	143	103	92
CRP (mg/dL)	3	0.27	0.48	1.29	0.21	0.9	0.02
Hb (g/dL)	15.9	15.1	9.8	11.4	16.4	13.4	13.3
Onset to balloon time	20 hours	6.5 hours	3 hours	Several days	2.5 hours	Several days	4.5 hours

**Figure 2:**
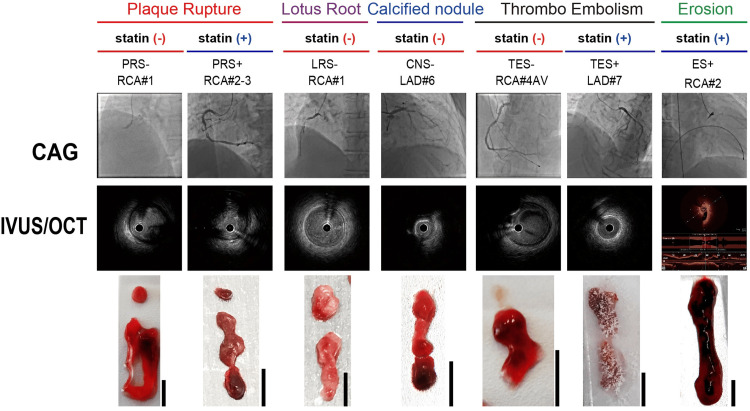
CAG, IVUS, and macroscopic classification of aspirated coronary thrombi Representative images of the culprit lesions obtained using CAG and IVUS, alongside macroscopic classifications of the aspirated coronary thrombi. Macroscopic images correspond to five thrombus types: PR, LR, CN, TE, and E. Scale bars (5 mm) are shown in each panel. Plaque rupture (IVUS): Fibrous cap disruption with a cavity communicating with the lumen. Lotus-root-like lesion (IVUS): Multiple intraluminal channels separated by septa, consistent with recanalized organized thrombus. Calcified nodule (IVUS): Protruding, irregular calcified mass extending into the lumen with acoustic shadowing. Plaque erosion (OCT): Attached thrombus over an intact fibrous cap without evidence of cap disruption. Thromboembolism (TE): Culprit-lumen thrombus without intravascular imaging evidence of an underlying atherosclerotic lesion, supported by angiographic and/or clinical features suggestive of coronary embolism. CAG: coronary angiography, IVUS: intravascular ultrasound, PR: plaque rupture, LR: lotus-root-like lesion, CN: calcified nodule, TE: thromboembolism, E: plaque erosion

Based on coronary angiography and intravascular ultrasound (IVUS) findings, thrombi were classified into five pathological categories: plaque rupture (PR), lotus-root-like organized thrombus (LR), calcified nodule (CN), thromboembolism (TE), and plaque erosion (E) (Figure 2, Table 1). For descriptive purposes, patients were categorized as statin-treated or statin-naïve according to medication information available at presentation. Statin-treated patients were defined as those receiving any statin therapy at the time of admission, whereas statin-naïve patients had no documented statin use at presentation. Macroscopic examination further classified aspirated thrombi into red thrombi (erythrocyte- and fibrin-rich), white thrombi (platelet-dominant), and mixed thrombi (Figure 2).

White thrombi were observed in two cases: a statin-naïve calcified nodule (CNS-) and a statin-naïve lotus-root-like lesion (LRS-). These macroscopic and IVUS-based classifications provided the structural framework for subsequent molecular and elemental analyses using mass spectrometry imaging and EDS-based elemental mapping (Figure 1). Clinical laboratory parameters showed substantial inter-individual variability across thrombus categories. The CNS- case exhibited elevated triglycerides (248 mg/dL), LDL-C (173 mg/dL), and CRP (1.29 mg/dL) in the absence of lipid-lowering therapy. ACS events were observed in both statin-treated and statin-naïve patients. Among PR cases, one statin-treated patient (PRS+) showed LDL-C of 78 mg/dL and CRP of 0.27 mg/dL, whereas one statin-naïve patient (PRS-) showed LDL-C of 144 mg/dL and CRP of 3 mg/dL; both presented with angiographically confirmed intracoronary thrombotic occlusion. A statin-treated TE case (TES+) showed LDL-C of 103 mg/dL and CRP of 0.9 mg/dL. Anemia was observed in the CNS- and LRS- cases. The time from symptom onset to coronary reperfusion (onset-to-balloon time) varied widely among patients and was therefore summarized descriptively and not incorporated into comparative analyses. Collectively, these findings highlight the clinical heterogeneity of the study cohort and provide contextual background for the subsequent molecular and elemental characterization, without implying causal relationships.

Molecular localization in thrombi by MALDI-mass spectrometry imaging (MALDI-MSI)

**Figure 3:**
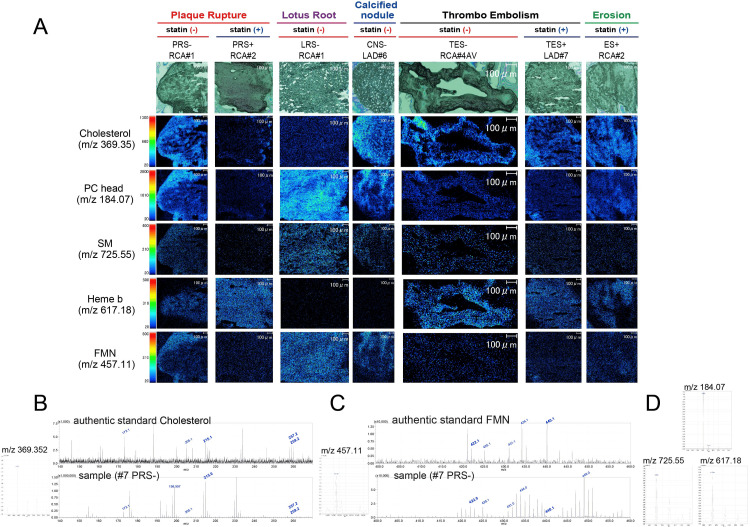
Spatial distribution of representative molecules visualized by MALDI-MSI MALDI-MSI visualized the spatial distributions of representative molecules across thrombus sections, including cholesterol, PC, SM, FMN, and heme b (annotated) (Figure 3A). The representative detected ions were cholesterol (m/z 369.35, Figure 3B), FMN (m/z 457.11, Figure 3C), the PC headgroup (m/z 184.07), putative SM (m/z 725.55), and heme b (m/z 617.18) (Figure 3D). Cholesterol signals were observed across all thrombus types, with heterogeneous distribution patterns among pathological categories. In PR, higher cholesterol signal intensities were observed in statin-naïve cases compared with statin-treated cases, whereas LR lesions exhibited relatively low cholesterol signals. PC and SM showed variable distribution patterns across PR and LR lesions. Heme b was prominently detected in PR and TE, consistent with erythrocyte-rich thrombi. FMN displayed lesion-type-dependent spatial distribution patterns across PR, LR, CN, TE, and E (lines 1-5). MALDI-MSI: matrix-assisted laser desorption/ionization mass spectrometry imaging, PC: phosphatidylcholine, SM: sphingomyelin, FMN: flavin mononucleotide, PR: plaque rupture, LR: lotus root, TE: thromboembolism, CN: calcified nodule, E: plaque erosion

Molecular annotations were classified as either putatively identified or putatively assigned.Putatively identified annotations were supported by concordant accurate mass measurements and matching MS/MS fragmentation patterns obtained from tissue sections and authentic standards, whereas putatively assigned annotations were based on accurate mass matching and reference to prior literature. MALDI mass spectrometry imaging enabled spatial visualization of molecular distributions within thrombus sections. Representative detected ions included cholesterol (m/z 369.35; putatively identified; Figure 3B, Table 2), the phosphatidylcholine (PC) headgroup (m/z 184.07; putatively assigned; Figure 3D, Table 2), putative sphingomyelin (SM; m/z 725.55; putatively assigned; Figure 3D, Table 2), flavin mononucleotide (FMN; m/z 457.11; putatively identified; Figure 3C, Table 2), and heme b (m/z 617.18; putatively assigned; Figure 3D, Table 2). As inherent to MSI-based annotation, the presence of structural or stereoisomers with identical or closely related m/z values cannot be completely excluded.

**Table 2:** Molecular annotations of representative ions detected by MALDI mass spectrometry imaging Table 2 summarizes representative ions detected by MALDI mass spectrometry imaging, including observed and theoretical m/z values, mass error (Δppm), proposed molecular annotations, ion species, identification levels, and MS/MS fragment ions when available. Molecular annotations were categorized as either putatively identified or putatively assigned. Putatively identified annotations were supported by concordant accurate mass measurements and matching MS/MS fragmentation patterns obtained from tissue sections and authentic standards. Putatively assigned annotations were based on accurate mass matching, consideration of ion species, database searches, and reference to prior literature, without direct MS/MS confirmation using standards. Cholesterol (m/z 369.35, [M-H₂O+H]⁺) and FMN (m/z 457.11, [M+H]⁺) were classified as putatively identified based on agreement of both precursor mass and MS/MS fragment ions with those of authentic standards. The phosphatidylcholine headgroup (m/z 184.07, [M+H]⁺), heme b (m/z 617.18, [M+H]⁺), and sphingomyelin (m/z 725.55, SM 34:1;O₂, [M+Na]⁺) were classified as putatively assigned based on accurate mass matching and established reports in the literature and databases. MALDI: matrix-assisted laser desorption/ionization, FMN: flavin mononucleotide, PC: phosphatidylcholine, SM: sphingomyelin

-	Observed m/z	Theoretical m/z	Delta	Δppm	Molecular annotation	Ion species	-	Identification level	MS/MS fragment
Cholesterol	369.35150	369.35158	0.00008	-0.210	Cholesterol (cholest-5-en-3-ol)	[M-H_2_O+H]^+^	C_27_H_46_O	Putatively identified	173.1, 205.1, 215.1, 257.2, 259.2
PC head	184.07360	184.07332	-0.00028	1.516	PC head	[M+H]^+^	C_5_H_14_NO_4_P	Putatively assigned	-
SM	725.55160	725.55680	0.00520	-7.167	SM 34:1;O2	[M+Na]^+^	C_39_H_79_N_2_O_6_PNa	Putatively assigned	-
Heme b	617.18020	617.18457	0.00437	-7.087	Heme b (Heme)	[M+H]^+^	C_34_H_32_FeN_4_O_4_	Putatively assigned	-
FMN	457.10910	457.11189	0.00279	-6.105	Flavin mononucleotide	[M+H]^+^	C_17_H_21_N_4_O_9_P	Putatively identified	422.1, 425.1, 434.1, 440.1

Cholesterol signals were detected across all thrombus categories, consistent with prior observations that cholesterol is commonly present in coronary thrombi. Within PR lesions, higher cholesterol signal intensities were observed in statin-naïve cases compared with statin-treated cases, with preferential localization toward the thrombus periphery. In contrast, TE and E lesions exhibited relatively abundant cholesterol signals with a diffuse intrathrombus distribution, irrespective of statin exposure. LR lesions showed comparatively lower cholesterol signal intensities, including in statin-naïve cases (Figure 3A, line 1). PC-derived ions were detected across all thrombus types and were particularly prominent in LR lesions. Higher PC signal intensities were observed in statin-naïve LR cases compared with statin-treated cases (Figure 3A, line 2). Putative SM signals varied among thrombus types and were generally lower in statin-treated cases, whereas PR lesions without statin therapy exhibited relatively higher SM signal intensities (Figure 3A, line 3). Heme b signals were strongly detected in PR and TE lesions, corresponding to red thrombus phenotypes, and were reduced in LR and CN lesions, which were predominantly classified as white thrombi (Figure 3A, line 4). FMN exhibited heterogeneous distribution patterns, with higher signals observed in PR lesions without statin therapy, as well as in LR and CN lesions, and lower signals in TE lesions (Figure 3A, line 5). These observations describe spatial distribution patterns of molecular signals within thrombus sections and represent associations within the studied cohort; they do not imply mechanistic relationships or causal effects.

Distributional analysis of MSI signal intensities

**Figure 4:**
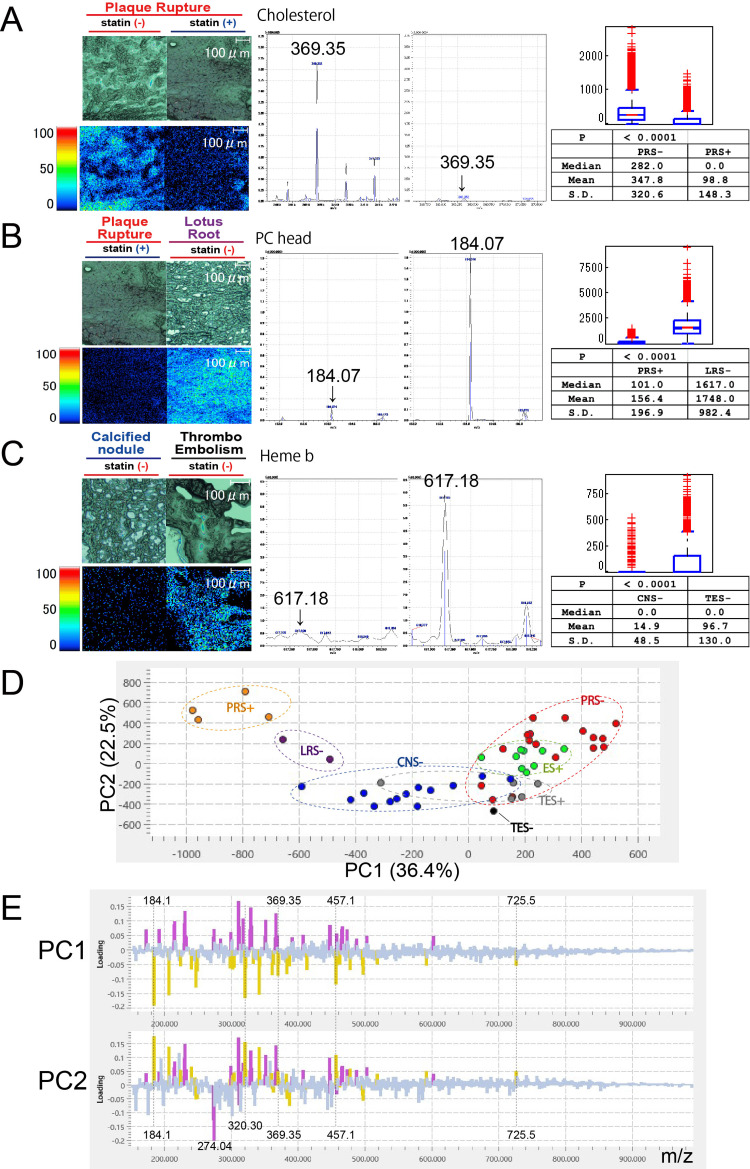
Distributional analysis of MSI signal intensities and exploratory PCA across IVUS-defined thrombus types MSI-derived spectral intensities of cholesterol, the PC headgroup, and heme b were compared between predefined lesion groups. Cholesterol intensities were higher in PRS- than in PRS+ thrombi (Figure 4A). PC headgroup signals were lower in PRS+ compared with LR thrombi (Figure 4B). Heme b intensity was lower in CNS- than in TES- thrombi, reflecting zero-inflated and sparsely distributed MSI signals (Figure 4C). PCA was performed on ROI-averaged spectra following TIC normalization and Pareto scaling. Each data point represents an individual ROI (200 μm × 200 μm). The first principal component (PC1) and second principal component (PC2) accounted for 36.4% and 22.5% of the total variance, respectively (Figure 4D). The PCA score plot (Figure 4D) was generated as an unsupervised, exploratory visualization to summarize global molecular variability in the MSI data and to assess potential trends across IVUS-defined thrombus categories using MSI-derived m/z variables and normalized ion intensities. The PCA loading vector (Figure 4E) illustrates representative molecular features contributing to variance along PC1 and PC2. Lipid-associated ions, including cholesterol (m/z 369.3), the phosphatidylcholine headgroup (m/z 184.1), and flavin mononucleotide-related signals (m/z 457.1), are shown as examples. The magnitude and direction of each loading vector indicate relative covariance with the distribution of samples in the PCA space and do not imply statistical significance, absolute quantitative changes, or causal relationships. Accordingly, PCA results should be interpreted cautiously and regarded as hypothesis-generating rather than confirmatory. MSI: mass spectrometry imaging, PCA: principal component analysis, IVUS: intravascular ultrasound, PC: phosphatidylcholine, LR: lotus root, ROI: region of interest, TIC: total ion current

Spectral intensities for selected representative molecules (cholesterol, the PC headgroup, and heme b) were statistically compared between predefined groups using pixel- and ROI-level MSI data (Figure 4).

These comparisons were performed to assess distributional and pattern-level differences in signal intensities rather than patient-level effects. For cholesterol, PRS- thrombi exhibited higher spectral intensities than PRS+ thrombi. The PRS- group showed a median intensity of 282.0 (mean: 347.8, SD: 320.6), whereas the PRS+ group showed a median of 0.0 (mean: 98.8, SD: 148.3) (Figure 4A).　For the PC headgroup, PRS+ thrombi showed lower signal intensities compared with LRS- thrombi. The PRS+ group showed a median intensity of 101.0 (mean: 156.4, SD: 196.9), whereas the LRS- group showed a median of 1617.0 (mean: 1748.0, SD: 982.4) (Figure 4B).

For heme b, CNS- thrombi exhibited lower signal intensities than TES- thrombi. Both groups had a median intensity of 0.0 due to a high proportion of zero-intensity pixels. However, TES- thrombi showed a greater frequency and magnitude of non-zero pixels, reflected by higher mean intensity (96.7 versus 14.9) and standard deviation (130.0 versus 48.5). This zero-inflated distribution resulted in a significant difference detected by the Mann-Whitney U-test (p < 0.0001) despite identical median values (Figure 4C).　These statistical comparisons describe differences in pixel- and ROI-level signal distributions within the analyzed thrombus sections. Given the zero-inflated nature of MSI data and the lack of independence among pixels and ROIs, p-values should be interpreted cautiously and should not be regarded as direct evidence of between-patient biological differences.

Principal component analysis (PCA) was performed to explore global spectral variation in the MSI dataset. PCA of ROI-averaged spectra showed that the first principal component (PC1) accounted for 36.4% of the total variance, while the second principal component (PC2) explained an additional 22.5%. Thus, a substantial proportion of overall spectral variability was captured by the first two principal components. The PCA score plot based on PC1 and PC2 showed separation patterns among samples, supporting the use of two-dimensional PCA visualization to summarize major trends in the MSI data (Figure 4D). As prespecified, PCA was used solely for exploratory visualization, and no statistical inference was derived from the PCA results. Inspection of PCA loading vectors indicated that lipid-associated ions contributed prominently to variance along PC1 and PC2. In particular, cholesterol (m/z 369.35), the phosphatidylcholine headgroup (m/z 184.1), and flavin mononucleotide-related signals (m/z 457.1) contributed substantially to the variance captured by both principal components (Figure 4E). The directionality of loading vectors reflects relative covariance with sample distribution in the PCA space rather than absolute increases or decreases in molecular abundance.

Elemental analysis by scanning electron microscopy (SEM) and energy-dispersive X-ray spectroscopy (EDS)

**Figure 5:**
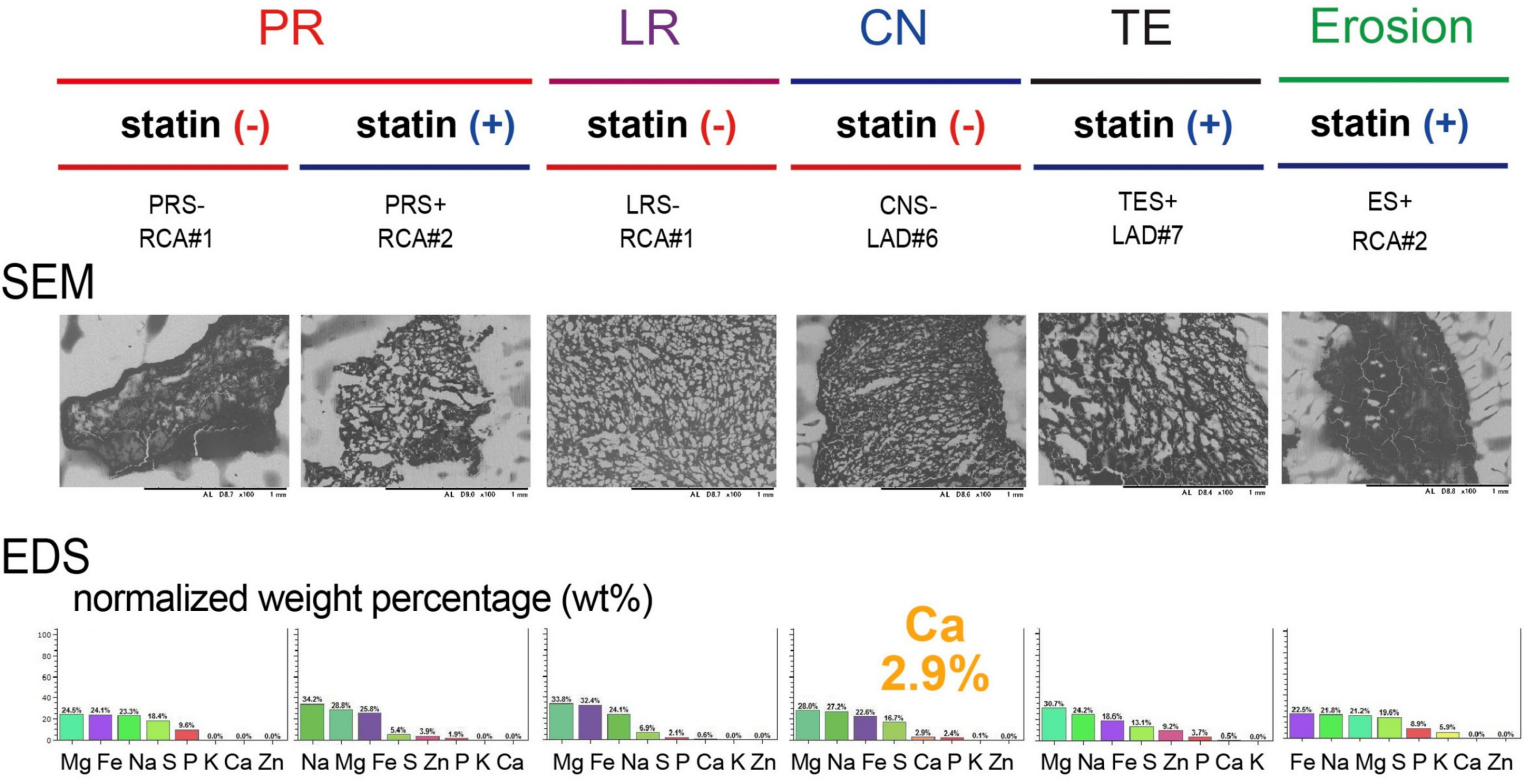
Structural and elemental characterization of thrombi by SEM and EDS Adjacent serial sections corresponding to those used for MALDI-MSI were examined by SEM/EDS. SEM revealed distinct ultrastructural features across thrombus types, including laminar and fibrous architectures in PR and E lesions, and a characteristic reticular architecture in LR lesions. Elemental mapping by EDS showed prominent calcium enrichment in CN lesions, consistent with their pathological classification. Additional elements, including Na, Mg, P, S, K, Zn, and Fe, exhibited heterogeneous and lesion-dependent spatial distributions across thrombus types. SEM/EDS findings are presented as semi-quantitative and are interpreted in conjunction with MSI-based molecular localization, providing complementary structural and elemental context rather than absolute elemental quantification. Elemental values are shown as normalized weight percentages (wt%) derived from EDS spectra. SEM: scanning electron microscopy, EDS: energy-dispersive X-ray spectroscopy, MALDI-MSI: matrix-assisted laser desorption/ionization mass spectrometry imaging, PR: plaque rupture, E: plaque erosion, LR: lotus root, CN: calcified nodule

Adjacent serial sections corresponding to those used for MALDI-MSI were analyzed by SEM/EDS (Figure 5). SEM revealed diverse ultrastructural morphologies across thrombus types, including laminar, fibrous, reticular, and heterogeneous architectures. Laminar structures were predominantly observed in PR and plaque erosion E lesions, whereas LR lesions exhibited a characteristic reticular architecture consistent with organized thrombus formation (Figure 5).

Elemental mapping by EDS showed prominent calcium enrichment in CN lesions, consistent with their pathological classification. Additional elements, including Na, Mg, P, S, K, Zn, and Fe, were detected with variable spatial distributions across thrombus types (Figure 5). These elemental signals showed heterogeneous localization patterns within thrombus regions rather than uniform distribution. Elemental data derived from EDS were interpreted in a semi-quantitative manner. Normalized weight percentages (wt%) were used to compare relative elemental composition across regions analyzed under identical acquisition conditions; however, these values do not represent absolute elemental concentrations. Accordingly, SEM/EDS findings are presented as descriptive, providing complementary structural and elemental context to the molecular distributions observed by MSI, rather than absolute elemental quantification.

## Discussion

In this study, we focused on representative molecular signals associated with major pathophysiological axes of thrombus formation, including lipid burden and membrane microdomain composition (cholesterol and sphingomyelin (SM)) [11-13], membrane-related metabolites (phosphatidylcholine (PC) and flavin mononucleotide (FMN)), markers of erythrocyte-rich thrombus phenotype (heme b), and residual lipid or energy storage [14]. Using an IVUS-based classification framework, we compared spatial, molecular, and elemental distribution patterns across distinct thrombus types and examined how these patterns varied among pathological categories. These analyses were intended to characterize distributional features within thrombus sections rather than to infer causal mechanisms.

Cholesterol was detected across all thrombus types, indicating that lipid components are a common feature of coronary thrombi in acute coronary syndrome. In plaque rupture (PR), higher cholesterol signal intensities were observed in statin-naïve cases compared with statin-treated cases. This difference represents an observed association between clinical background and intrathrombus molecular patterns rather than evidence of a direct therapeutic effect of statin therapy on thrombus composition. Notably, ACS occurred in both statin-treated and statin-naïve patients, including cases with low circulating LDL-C and low CRP values. These observations highlight the heterogeneity of thrombus biology and underscore that residual cardiovascular risk can persist despite lipid-lowering therapy [4-6].

Lotus-root-like (LR) lesions identified by IVUS exhibited morphological features consistent with chronically organized thrombi, including multiple interconnected channels separated by septa [10]. In these lesions, cholesterol signals were relatively low even in statin-naïve cases. This pattern may reflect differences in thrombus age, degree of organization, and extent of plaque involvement compared with plaque rupture (PR), rather than differences in systemic lipid status alone.

In contrast, thromboembolism (TE) and plaque erosion (E) lesions showed relatively high and diffusely distributed cholesterol signals irrespective of statin exposure. These observations suggest that intrathrombus cholesterol accumulation likely reflects multiple contributing factors, such as incorporation of circulating lipoproteins, erythrocyte membrane components, and local hemodynamic conditions, rather than solely local plaque lipid content.

PC-derived ions were detected across all thrombus types and were particularly prominent in lotus-root-like (LR) lesions. In organized thrombi, increased cellularity and extracellular matrix remodeling may be associated with a greater abundance of membrane-related lipid components, which could contribute to elevated PC signals. Differences in PC signal intensity between statin-treated and statin-naïve cases were observed in some pathological categories; however, given the limited cohort size and the imbalance in statin exposure, these observations should be interpreted as descriptive and context-dependent rather than as evidence of biological modulation of thrombus composition by statin therapy.

SM, a key component of lipid rafts [11-13], exhibited variable distribution patterns among thrombus types. Reduced SM signals were observed in several statin-treated cases, whereas relatively higher SM signals were detected in statin-naïve PR lesions. These observations may reflect differences in cellular composition, membrane turnover, or the contribution of plaque-derived material within thrombi, rather than a direct effect of statin therapy. Further studies integrating lipid raft-associated proteins, such as caveolin and flotillin, together with immune cell markers, will be required to clarify the biological relevance of SM distribution during thrombus formation and maturation.

Heme b showed strong signals in red thrombi, such as PR and TE, and lower signals in predominantly white thrombi, such as LR and CN. This distribution closely matched macroscopic thrombus appearance, indicating that MSI effectively captures erythrocyte-rich versus platelet-dominant thrombus phenotypes at the molecular level. In TE, which is frequently associated with blood stasis and erythrocyte-rich thrombus formation, elevated heme b signals likely reflect increased red blood cell content. In contrast, lower heme b signals were observed in LR and CN lesions, and some of these cases were accompanied by anemia, suggesting that systemic hematologic status may influence intrathrombus molecular composition. Combined analyses of red blood cell degradation products and iron metabolism-related molecules may provide additional insights into thrombus composition and temporal evolution. Furthermore, future studies incorporating enzymes involved in neutral lipid metabolism, including adipose triglyceride lipase and hormone-sensitive lipase, may help clarify lipid metabolic differences between acute thrombi and chronically organized thrombi.

FMN exhibited heterogeneous distribution patterns across thrombus types. In PR lesions, higher FMN signals were observed in statin-naïve cases compared with statin-treated cases, whereas LR and CN lesions showed relatively elevated FMN signals. These patterns may reflect differences in cellular density or metabolic state within thrombi; however, FMN assignment and interpretation remain provisional and should be regarded as hypothesis-generating rather than definitive. Further validation using MS/MS-confirmed molecular identification and complementary assessments of mitochondrial and metabolic markers, such as mitochondrial DNA content, ATP/ADP ratios, and enzymes involved in oxidative phosphorylation, will be necessary to clarify the biological significance of FMN-related signals [15].

SEM analysis revealed characteristic structural patterns corresponding to thrombus pathology and the degree of organization. Laminar architectures were predominantly observed in PR and E lesions, whereas LR lesions exhibited a reticular structure, consistent with IVUS-based classification [10]. Calcified nodules showed prominent calcium enrichment on EDS mapping, directly reflecting their defining pathological feature.

Additional elements, including Mg and P, which are associated with mineralization-related processes, and Fe, which is related to erythrocyte content, showed heterogeneous and thrombus-type-dependent distributions. Notably, the spatial concordance between Fe detected by EDS and heme b signals observed by MSI supports the complementary nature of elemental and molecular imaging approaches. Together, these multimodal observations provide a descriptive framework for characterizing the structural, molecular, and elemental heterogeneity of coronary thrombi, without implying mechanistic or causal relationships.

This study has several limitations. First, this was a real-world observational study of patients with acute coronary syndrome, many of whom were transferred from community hospitals. As a result, precise information regarding the duration of statin therapy, cumulative exposure, and medication adherence prior to the index event could not be objectively determined. Accordingly, statin-related findings should be interpreted as descriptive and hypothesis-generating rather than indicative of causal or dose-dependent effects. The terms “statin-treated” and “statin-naïve” were used solely as operational definitions based on medication status at presentation and do not reflect long-term exposure, treatment duration, or adherence. In addition, the distribution of statin-treated and statin-naïve patients was unbalanced across thrombus types, and statin exposure was treated as a binary variable without detailed information on dose or duration. Therefore, any associations related to statin use should be interpreted as reflecting clinical context rather than direct effects on thrombus composition.

Second, the small sample size and the use of pixel- and region-of-interest-level analyses limit statistical power and preclude robust patient-level inference, raising the possibility of pseudo-replication. Consequently, statistically significant p-values should be interpreted with caution and regarded as supporting spatial or pattern-level differences rather than definitive between-patient effects.

Third, although MALDI mass spectrometry imaging provides high spatial resolution and enables integrated molecular and elemental visualization, quantitative comparisons across samples and multivariate analyses such as principal component analysis were conducted solely within an exploratory, hypothesis-generating framework. These approaches require further methodological standardization, validation, and orthogonal confirmation. Future studies incorporating larger, prospectively enrolled cohorts with balanced clinical backgrounds, together with multimodal validation using immunohistochemistry, functional assays, and circulating biomarkers, will be essential to determine whether spatial molecular and elemental profiling of coronary thrombi can ultimately contribute to clinically meaningful classification or risk stratification frameworks.

In addition, PCA was used exclusively as an unsupervised, exploratory tool to visualize global spectral variation in the MSI data. Accordingly, PCA results should be interpreted descriptively and were not intended for statistical inference, classification, or predictive modeling. PCA was performed on ROI-averaged spectra, which may attenuate pixel-level heterogeneity within individual regions. This approach was adopted to reduce the influence of local noise and to ensure consistent comparisons across samples with variable tissue size and morphology. Finally, no cross-validation or robustness testing was applied to PCA, as the analysis was not used for hypothesis testing or model validation in this study.

## Conclusions

Within these limitations, we integrated molecular mapping by matrix-assisted laser desorption/ionization mass spectrometry imaging (MALDI-MSI) with structural and elemental characterization by scanning electron microscopy and energy-dispersive X-ray spectroscopy (SEM/EDS) to explore the molecular and elemental heterogeneity of coronary thrombi across intravascular ultrasound-defined pathological categories. Representative molecules, including cholesterol, phosphatidylcholine, sphingomyelin, heme b, triglycerides, and flavin mononucleotide, exhibited heterogeneous spatial distribution patterns that varied according to thrombus type and lesion characteristics. These molecular patterns were observed in the context of diverse clinical backgrounds, including differences in statin exposure and lesion organization stage, and should be interpreted as descriptive, spatially resolved associations rather than causal relationships. Elemental analysis further revealed pathology-consistent features, such as prominent calcium accumulation in calcified nodules, providing complementary structural and compositional context to the molecular imaging findings.　Overall, this exploratory, hypothesis-generating study provides an initial framework for linking imaging-based thrombus classification with spatial molecular and elemental features in situ. Further validation in larger, prospectively enrolled cohorts, together with orthogonal and quantitative approaches, will be required to determine whether integrated molecular and elemental profiling of coronary thrombi can ultimately contribute to clinically meaningful classification schemes or inform future diagnostic and therapeutic strategies in acute coronary syndrome.
